# Lipid-mediated resolution of inflammation and survival in amyotrophic lateral sclerosis

**DOI:** 10.1093/braincomms/fcae402

**Published:** 2025-01-15

**Authors:** Ozlem Yildiz, Guy P Hunt, Johannes Schroth, Gurleen Dhillon, Thomas P Spargo, Ammar Al-Chalabi, Sulev Koks, Martin R Turner, Pamela J Shaw, Sian M Henson, Alfredo Iacoangeli, Andrea Malaspina

**Affiliations:** Neuromuscular Department, Motor Neuron Disease Centre, Queen Square Institute of Neurology, University College London, London WC1N 3BG, UK; Neuroscience and Trauma, The Blizard Institute, Queen Mary University of London, London E1 2AT, UK; Department of Biostatistics and Health Informatics, Institute of Psychiatry, Psychology and Neuroscience, King’s College London, London SE5 8AF, UK; Department of Basic and Clinical Neuroscience, Maurice Wohl Clinical Neuroscience Institute, Institute of Psychiatry, Psychology and Neuroscience, King’s College London, London SE5 9RT, UK; Perron Institute for Neurological and Translational Science, Research Institute in Nedlands, WA 6009, Australia; Centre for Molecular Medicine and Innovative Therapeutics, Murdoch University, Perth 6150, Australia; Translational Medicine and Therapeutics, William Harvey Research Institute, Queen Mary University of London, London EC1M 6BQ, UK; Neuroscience and Trauma, The Blizard Institute, Queen Mary University of London, London E1 2AT, UK; Department of Basic and Clinical Neuroscience, Maurice Wohl Clinical Neuroscience Institute, Institute of Psychiatry, Psychology and Neuroscience, King’s College London, London SE5 9RT, UK; Department of Basic and Clinical Neuroscience, Maurice Wohl Clinical Neuroscience Institute, Institute of Psychiatry, Psychology and Neuroscience, King’s College London, London SE5 9RT, UK; Maurice Wohl Clinical Neuroscience Institute, King’s College Hospital, London SE5 9RS, UK; Perron Institute for Neurological and Translational Science, Research Institute in Nedlands, WA 6009, Australia; Centre for Molecular Medicine and Innovative Therapeutics, Murdoch University, Perth 6150, Australia; Nuffield Department of Clinical Neurosciences, University of Oxford, Oxford OX3 7JX, UK; Sheffield Institute for Translational Neuroscience, University of Sheffield, Sheffield S10 2HQ, UK; Translational Medicine and Therapeutics, William Harvey Research Institute, Queen Mary University of London, London EC1M 6BQ, UK; Department of Biostatistics and Health Informatics, Institute of Psychiatry, Psychology and Neuroscience, King’s College London, London SE5 8AF, UK; Department of Basic and Clinical Neuroscience, Maurice Wohl Clinical Neuroscience Institute, Institute of Psychiatry, Psychology and Neuroscience, King’s College London, London SE5 9RT, UK; National Institute for Health Research Biomedical Research Centre and Dementia Unit at South London and Maudsley NHS Foundation Trust and King’s College London, London SE5 8AF, UK; Neuromuscular Department, Motor Neuron Disease Centre, Queen Square Institute of Neurology, University College London, London WC1N 3BG, UK; Neuroscience and Trauma, The Blizard Institute, Queen Mary University of London, London E1 2AT, UK

**Keywords:** neuroinflammation, amyotrophic lateral sclerosis, specialized pro-resolving mediators, resolvins, GPR32/GPR18

## Abstract

Neuroinflammation impacts on the progression of amyotrophic lateral sclerosis (ALS), a fatal neurodegenerative disorder. Specialized pro-resolving mediators trigger the resolution of inflammation. We investigate the specialized pro-resolving mediator blood profile and their receptors’ expression in peripheral blood mononuclear cells in relation to survival in ALS. People living with ALS (pwALS) were stratified based on bulbar versus limb onset and on key progression metrics using a latent class model, to separate faster progressing from slower progressing ALS. Specialized pro-resolving mediator blood concentrations were measured at baseline and in one additional visit in 20 pwALS and 10 non-neurological controls (Cohort 1). Flow cytometry was used to study the GPR32 and GPR18 resolvin receptors’ expression in peripheral blood mononuclear cells from 40 pwALS and 20 non-neurological controls (Cohort 2) at baseline and in two additional visits in 17 pwALS. Survival analysis was performed using Cox proportional hazards models, including known clinical predictors and GPR32 and GPR18 mononuclear cell expression. Differential expression and linear discriminant analyses showed that plasma resolvins were able to distinguish phenotypic variants of ALS from non-neurological controls. RvE3 was elevated in blood from pwALS, whilst RvD1, RvE3, RvT4 and RvD1_n-3 DPA_ were upregulated in A-S and RvD2 in A-F. Compared to non-neurological controls, GPR32 was upregulated in monocytes expressing the active inflammation-suppressing CD11b^+^ integrin from fast-progressing pwALS, including those with bulbar onset disease (*P* < 0.0024), whilst GPR32 and GPR18 were downregulated in most B and T cell subtypes. Only GPR18 was upregulated in naïve double positive Tregs, memory cytotoxic Tregs, senescent late memory B cells and late senescent CD8^+^ T cells from pwALS compared to non-neurological controls (*P* < 0.0431). Higher GPR32 and GPR18 median expression in blood mononuclear cells was associated with longer survival, with GPR32 expression in classical monocytes (hazard ratio: 0.11, *P* = 0.003) and unswitched memory B cells (hazard ratio: 0.44, *P* = 0.008) showing the most significant association, along with known clinical predictors. Low levels of resolvins and downregulation of their membrane receptors in blood mononuclear cells are linked to a faster progression of ALS. Higher mononuclear cell expression of resolvin receptors is a predictor of longer survival. These findings suggest a lipid-mediated neuroprotective response that could be harnessed to develop novel therapeutic strategies and biomarkers for ALS.

## Teaser

Yildiz *et al*. describe the blood profile of specialized lipid mediators involved in inflammation resolution and of GPR32/GPR18 membrane receptors in blood mononuclear cells from patients with amyotrophic lateral sclerosis with a fast and slow rate of disease progression. Plasma resolvins were able to distinguish phenotypic variants of amyotrophic lateral sclerosis, and higher GPR32/GPR18 median expression was associated with longer survival.

## Graphical Abstract

**Graphical Abstract:**
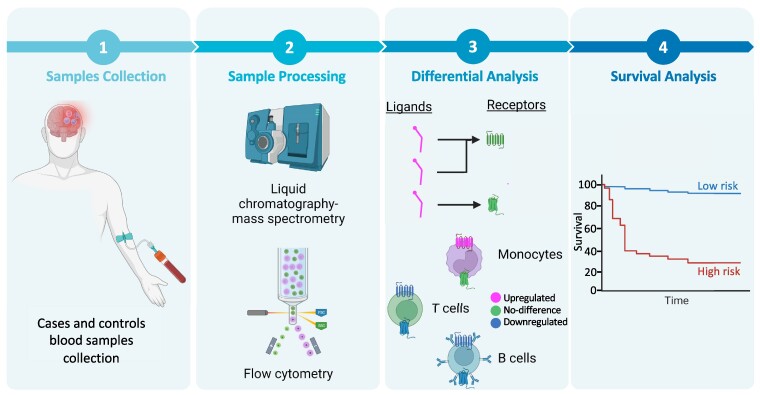

## Introduction

In amyotrophic lateral sclerosis (ALS), a clinically heterogeneous and fatal neurodegenerative disorder, the rate of motor cell loss is likely to determine the tempo of clinical progression. Adding to the pathological and clinical heterogeneity of ALS, experimental data have recently emerged depicting a state of immunological dysregulation.^1^ The appearance of pro-inflammatory peripheral lymphocytes and monocytes in ALS is observed in association with spinal cord and brain infiltration by macrophages and T cells, and the presence of activated microglia in affected tissues.^2^ A hallmark of ALS immunopathology is the loss of T regulatory/cells (Tregs), known to maintain a state of self-tolerance, prevent autoimmunity and keep chronic inflammation in check.^3^ ALS immunological dysregulation is also thought to be enhanced by genetic mutations linked to the familial form of the disease, including those involving *SOD1*, *TARDBP* and *C9orf72* genes.^3^

Neuroinflammation is a self-limiting response to antigens that are formed during infections, are derived from tissue injury or from the altered conformation, or aggregation, of proteins. Neuroinflammation acts as a protective mechanism that preserves the normal structure and function of the brain. In ALS, an initial, circumscribed and protective response (M2) is thought to be followed by a more extended and sustained pro-inflammatory reaction (M1), both in areas of neurodegeneration and systemically.^1^ In support of this hypothesis, we have recently shown increased frequencies of senescent CD4^+^CD27-CD57^+^ T cells, memory CD8^+^ T and late memory B cells, in blood from people living with ALS (pwALS). We have detected the systemic elevation of these senescent lymphocytes that sustain chronic inflammation in faster progressing ALS (A-F) and in ALS individuals with bulbar involvement (A-B). A higher frequency of late memory B cells is a predictor of shorter survival.^4^ We have also reported that higher levels of peripheral monocytes expressing the inflammation-suppressing active CD11b beta2 integrin are linked to better clinical outcomes in ALS.^5^

The attenuation and resolution of chronic inflammation are mediated by different converging molecular pathways, including the effect of specialized pro-resolving mediators (SPMs). These pro-resolving lipids are biosynthesised from ω-3 and ω-6 polyunsaturated fatty acids.^6-8^ SPMs are grouped into four chemical families: arachidonic acid (AA)-derived lipoxins (LXA4 and LXB4), eicosapentaenoic acid (EPA)-derived resolvins (RvE1–RvE4),^9^ docosahexaenoic acid (DHA)-derived resolvins (RvD1–RvD6), protectins (PD1/NPD1 and PDX) and maresins (MaR1 and MaR2).^10^ Recent experimental evidence mostly obtained from animal models highlights the effects mediated by resolvins on the resolution of inflammation and the biological mechanisms of neurodegenerative diseases such as Alzheimer’s disease,^11-13^ Parkinson’s disease,^14^ frontotemporal dementia^15^ and ALS.^16,17^ The concentration of DHA-derived maresins and resolvins is increased in CSF from frontotemporal dementia individuals with a *C9orf72* expansion.^15^ Critical to the immune dysregulation described in ALS, it has been shown that RvD1 can inhibit inflammatory macrophages and prevent phagocytosis of healthy neurons in the spinal cord.^16^ However, the extent of inflammation resolution and the way immunological processes leading to neuronal cells’ demise are modified by these low abundance endogenous lipid mediators is far from being understood.

The pro-resolving, anti-inflammatory effect of resolvins arises from the binding of these lipid mediators to specialized G protein-coupled receptors. Among these, both GPR32 and ALX/FPR2 resolvin receptors, expressed mostly in human leucocytes and adipose tissue, mediate the actions of RvD1–RvD6, inducing Treg differentiation,^18^ maintaining an immune-tolerogenic and anti-inflammatory state, and reducing microglia and astrocyte activation.^19^ RvD2’s effect depends on the GPR18 receptor whilst RvD1, RvD3, RvD5 and AT-RvD3 have been described to bind to GPR32.^20,21^ Both RvD1 and RvD2 promote macrophage-mediated phagocytosis and polarization towards a pro-resolution phenotype, preventing CD4^+^ T cell differentiation into Th1 and Th17 effectors and the production of harmful IFN-γ and IL-17.^22^ Receptor-mediated resolvin activity is thought to prevent neuronal cell death and promote functional recovery upon traumatic brain injury.^23^

Preventing chronic inflammation may therefore be a strategy for motor cell rescue and preservation in ALS. SPMs may be a critical target for therapeutics aiming at the mitigation of the chronic inflammatory processes that drive disease progression. Crucially, a better knowledge of the expression of resolvin receptors in circulating immune cells, including Tregs, may direct a SPM-based disease-modifying strategy in ALS. In this study, we first identify those SPMs that are detectable in blood from pwALS and investigate the correlation between the concentration of these lipid mediators and the ALS phenotype. In a separate ALS cohort, we characterize mononuclear cell subsets in blood, including macrophage precursors and senescent lymphocytes, to map the expression of relevant SPM receptors. We test the hypothesis that SPM-based inflammation resolution through critical immune cell receptors is a determinant of disease progression in ALS.

## Materials and methods

### Participant demographics, clinical characteristics and outcome measures

PwALS and age-matched non-neurological controls (NNC) were retrospectively identified from the ALS Biomarkers Study (09/H0703/27) and A Multicentre Biomarker Resource Strategy in ALS (AMBRoSIA; 16/LO/2136). All participants provided written informed consent to be enrolled at, or shortly after, diagnosis. Neurological impairment of different functional domains for each ALS individual was scored using an established functional rating scale [e.g. ALS Functional Rating Scale Revised (ALSFRS-R); 0–48: the lower the score, the greater the neurological compromise].^24^

We categorized pwALS into faster (A-F) and slower (A-S) progressors using two approaches to minimize the bias inherent to the use of patients’ outcome measure. First, as previously reported, pwALS were categorized based on ΔFRS [the difference of ALSFRS-R approximated to 48 at disease onset (neurologically healthy) and of ALSFRS-R at baseline sampling divided by the time interval in months) in faster (ΔFRS > 0.5) and slower (ΔFRS < 0.5) progressors.^4^ Additionally, categorization was performed based on the assignment of pwALS across data-driven clusters from a latent class analysis (LCA) model derived using ALS clinical characteristics sampled in large international data sets.^25,26^ This model defines five subgroups of ALS, with Class 1 being the most frequent and characterized by the shortest diagnostic delay and disease duration, suggesting faster progression. Within the LCA method, A-F were people assigned to Class 1, and A-S included Classes 2–5; a more fine-grained classification would not be feasible with the sample sizes available for the present study. We then identified the approach that best separated A-F from A-S across several key progression metrics including disease duration, ΔFRS, diagnostic delay, age at onset, sex and site of onset. The progression categorization approach that better separated A-F from A-S was used in all subsequent analyses.

In A-B and limb onset ALS (A-L), clinical (and neurophysiological) features were confined to the bulbar and cervical/thoracic/lumbar regions, respectively, for at least 6 months from symptom onset. Survival was defined as the time interval between baseline and permanent assisted ventilation (≥22 h per day of non-invasive ventilation), tracheostomy or death. To evaluate the progression rate post-baseline, we subtracted the last recorded ALSFRS-R score from the baseline ALSFRS-R and divided it by the time interval (ALSFRS-R change, points/month). Participants with a medical history of autoimmune disease, recent head or spinal injury, or chronic and systemic inflammatory disorders were excluded from the study.

### Human plasma and peripheral blood mononuclear cell separation

Whole blood was collected by venepuncture in 3–4 EDTA blood tubes. Plasma was separated from heparinized blood and peripheral blood mononuclear cells (PBMCs) were isolated by stratifying ∼30 mL of diluted blood with an equal amount of Dulbecco’s phosphate-buffered saline on 15 mL of Lymphoprep™ (density gradient medium, STEMCELL Technologies), followed by centrifugation at 2000 rpm for 40 min at room temperature. Ten percent dimethyl sulfoxide freezing solution (Cell Signaling Technology) and foetal bovine serum (FBS) were used for cryopreservation. The period of PBMC immersion in liquid nitrogen and thawing for flow cytometry (FC) staining is defined as cryopreservation time (documented in days for each sample). In our previous work, we have shown that cryopreservation time does not affect the recovery of lymphocyte subsets and frequencies of lymphocyte subsets do not correlate to the storage time.^4^

### SPM analysis: chiral liquid chromatography–mass spectrometry/mass spectrometry

Lipid mediators including resolvins, maresins and protectins are derived from AA, EPA, N-3 docosapentaenoic acid (n-3 DPA) and DHA fatty acid metabolomes (Supplementary Fig. 1). These lipid mediators were separated from plasma using solid-phase extraction columns and analysed utilizing a QTrap 5500 operated in negative ionization mode for multiple reaction monitoring. Signatures of ion fragments for each molecule were obtained by testing isobaric monohydroxy fatty acid levels.^27^ Standard operating procedures for SPM extraction from plasma samples, for analysis and quantitation were applied according to published guidelines, and quality checks were undertaken using the framework for quality and reliability evaluation of targeted metabolomic assays.^28,29^ These guidelines follow a comprehensive review of methodologies that can also be found in Protocol Exchange, an open repository of community-contributed protocols, which are freely shared.^30^

### FC preparation and staining

Cryopreserved PBMCs were thawed at room temperature and re-suspended in 10 mL warm Roswell Park Memorial Institute (RPMI) 1640 medium, supplemented with 10% FBS and 1% penicillin streptomycin. The cell suspension was centrifuged at 483 g for 10 min at room temperature and the pellet re-suspended twice in RPMI 1640 medium. PBMCs (1–2 × 10^6^ cells per tube) were stained for 15 min with 100 μL of diluted viability dye zombie aqua (Biolegend®, 1:100 in Dulbecco's phosphate buffered saline). Fifty microlitres of diluted Fc Block (Human BD Fc Block™) was added for another 15 min to block potential non-specific antibody staining. The cell suspension was washed (with FC staining buffer at 400 g for 5 min). Then, unconjugated primary antibodies were added: (i) GPR32 (GeneTex, GTX108119, rabbit) or (ii) GPR18 (Novus, NBP2-24918, rabbit). As a second step, the conjugated antibodies for the (i) T cell panel [CD127 (HIL-7R-M21)-BV510, CD3 (Hit3a)-BV605, CD8 (RPA-T8)-BV650, CD25 (2A3 and M-A251)-BV786, CD45RO (UCHL1)-PE-CF594, CD4 (L200)-PerCP-Cy5.5], (ii) B cell panel [CD24 (ML5)-BV605, CD27 (M-T271)-BV650, CD19 (HIB19)-PE-CF594, IgD (IA6-2)-PerCP-Cy5.5], or (iii) monocyte panel [CD11b(ICRF44)-BV421, CD14(M5E2)-BV605, HLA-DR(G46-6)-BV650, CD16(3G8)—PE-CF594, CD11b activated(CBRM1/5)-PE-Cy7] or (iv) senescent T cell panel [CD4 (RPA-T4)-PE-CF594, CD8 (SK1)-PerCP, CD27 (0323)-BV421, CD45RA (HI100)-BV605, CCR7 (G0343H7)-PeCy7, CD28 (CD28.2)-BV785, KLRG1 (REA261)-PE] were incubated for 30 min in the dark at room temperature. Fifty microlitres of Brilliant Stain Buffer was complemented to mitigate possible staining artefacts. For T cell intracellular staining, the cell suspensions were washed, fixed (200 μL intracellular fixation buffer, eBioscience, 1:1 diluted with FC buffer) and permeabilized (1 mL of nuclear fix/perm buffer, eBioscience 00-5523-00). Next, cells were stained with two clones of FoxP3 (259D/C7 and PCH1010)-PE to improve Treg detection.^31^ Experiments were carried out using the NovoCyte® 13-colour flow cytometer configured with 405, 488 and 640 nm lasers for data acquisition and analysis. Supplementary Table 1 summarizes all markers used for FC analysis and abbreviations.

### SPM analysis data processing

Consensus guidelines for metabolomic studies, quality control and validation of the mass spectrometry (MS) chromatogram peaks for small molecules/lipidomic studies were followed. These included (i) matching retention time to synthetic or authentic standards, (ii) a clearly defined limit of detection (LOD) and limit of quantitation (LOQ) of the targeted assays, (iii) a discernible peak visible above baseline noise with a specified number of data points (5 or above) and (iv) LOQ of signal-to-noise ratio of at least 5:1.^29^ All peaks were checked to ensure they reached the specified signal-to-noise ratio as well as the required number of data points. Calibration curves were obtained for each mediator using synthetic compound mixtures at 0.78, 1.56, 3.12, 6.25, 12.5, 25, 50, 100 and 200 pg that gave linear calibration curves with *r*^2^ values of between 0.98 and 0.99.

We performed principal component analysis on the SPMs derived from the four major fatty acid metabolomes. However, we observed that the concentration of SPMs in blood was heavily dependent on the use of statins and on sample storage time. To overcome this limitation, we used a supervised dimensionality reduction technique, linear discriminant analysis (LDA), to identify SPM signals that distinguish ALS subpopulations from NNC based on SPMs’ blood expression. LDA was performed using the MASS package.^32^

To perform LDA and differential expression analysis, we first inverse hyperbolic sine (arcsinh) transformed the data set following commonly used omics analysis procedures.^33^ We normalized the data set and compensated for a strong mean variance trend using limma precision weights. Differential expression analyses were performed using GEOexplorer^34^ and the limma package.^35-37^ The following covariates were included in our differential expression analysis: age at visit, sex, ethnicity, site of onset, years from onset to visit, progression rates, statin use, sample storage time and years from the previous visit in our longitudinal analysis. Wald test *P*-values were false discovery rate adjusted via the Benjamini–Hochberg procedure.^38^

### FC data pre-processing: unsupervised clustering and resolvin receptor cell expression

Within the diffcyt package,^39^ we used the testDS_limma function for the detection of differentially expressed lipid mediators. The following covariates were included: age at visit, sex, ethnicity, site of onset, years from onset to visit and progression category. Wald test *P*-values were false discovery rate adjusted via the Benjamini–Hochberg procedure.^38^

Data pre-processing and unsupervised clustering were performed using R version 4.2.2^40^ following the workflow of Melsen *et al*.^41^ We hyperbolic arcsinh transformed the data using automated cofactors calculated from the FlowVS package.^42^ Normalization was applied using fdaNorm as implemented in the FlowStats package.^43^ We removed cells that exceeded the 99.9 and 0.01 quantiles of each marker as outliers. Markers were separated into ‘phenotypic markers’ (CD4, CD8, CD127, CD25, FoxP3, CD45RO, CD27, CD45RA, CD28, KLRG1, CCR7, CD27, CD24, IgD, CD11b, CD14, HLA-DR, CD16 and activated CD11b) and ‘functional markers’ (resolvin receptors: GPR32 and GPR18), with minimum–maximum scaling performed on the phenotypic markers.

PhenoGraph was used for clustering analysis^44^ whilst the FlowSOM algorithm^45^ was applied to validate PhenoGraph results. The number of clusters for the FlowSOM models was determined using the elbow method (Supplementary Fig. 3) and manual inspection of uniform manifold approximation and projection dimensionality reduction plots. We applied the PhenoGraph algorithm with 50 *k*-nearest neighbours. Phenotypically similar clusters were merged into cell populations based on their median marker expression to avoid over clustering. We utilized the uniform manifold approximation and projection dimensionality reduction technique on 100 000 randomly selected cells to visualize the cell populations.^46^ Using bootstrapping, we randomly selected 80% of our patients 100 times and categorized cell populations based on their median Jaccard similarity coefficient into very high stability (>0.85), high stability (0.85–0.75), moderate stability (0.75–0.60) and low stability (<0.60)^47,48^ (Supplementary Fig. 4).

### Longitudinal analyses

The diffcyt package^39^ testDS_limma function was used for the detection of differentially expressed functional markers. To detect meaningful differential regulation across time points, we utilized the blocking parameter to enable a paired *t*-test. We included the following covariates: age at visit, sex, ethnicity, site of onset, years from onset to visit, progression rate and years from the previous visit. *P-*values were false discovery rate adjusted via the Benjamini–Hochberg procedure.^38^

### Survival analyses

Cox proportional hazards models were used to investigate the association between survival, clinical and biological variables. First, a model was fitted using only clinical and demographic variables as predictors (sex, site of onset, ALSFRS-R score, age at onset and ΔFRS). The optimum set of variables was identified using a stepwise approach. This analysis was conducted with the survivor, survminer and MASS packages.^32,49,50^

We next analysed whether the resolvin receptors under investigations (GPR32 and GPR18) could improve the model fit. We derived median expression of each of GPR32 and GPR18 in various cell types (biological markers) identified via unbiased clustering with PhenoGraph. The biological markers were standardized to have a mean of 0 and SD of 1. Correlations between biological markers were calculated. We identified groups of correlated biological markers with hierarchical clustering, using the Ward method, along with manual inspection of correlation matrix plots. We compared Cox proportional hazards models including both clinical and biological predictors with a model constructed with clinical variables only using an ANOVA test. These models were fitted using one biological marker at a time to avoid overfitting and multicollinearity. The robust variance was calculated for all Cox proportional hazards models. Furthermore, we validated the results by repeating the analysis using cell types identified by the FlowSOM algorithm.

The Bonferroni correction was used to adjust probability (*p*) values and reduced a type I error in statistical tests.

### Ethics approval and consent to participate

Ethical approval for the studies was obtained for the ALS Biomarkers Study (East London Research Ethics Committee, London, UK—REC reference 09/H0703/27) and for A Multicentre Biomarker Resource Strategy In ALS (‘AMBRoSIA’, South-East Research Ethics Committee—16/LO/2136). Informed consent is available from all participants.

## Results

### Demographic and clinical characteristics

For the blood SPM analysis, we selected baseline and 12-month follow-up samples (second visit: V2) from a cohort of 20 pwALS, including 10 A-F and 10 A-S [Cohort 1: mean age 61.5 (SD ± 10.2) years, 35% female] and baseline samples from 10 NNC [mean age 61 (SD ± 11.4) years, 40% female; ALS Biomarkers Study]. For the resolvin receptor analysis of circulating mononuclear cells by FC, we used the same cohort of pwALS and NNC recently investigated, to study the blood expression of senescent blood lymphocytes in ALS progression (Cohort 2).^4^ This included 40 pwALS [mean age 63.9 years (SD ± 11.4), 52.5% female] and 20 age-matched and sex-matched NNC [mean age 60.4 years (SD ± 7.1), 50% female] (Table 1). Forty-five per cent of pwALS from Cohort 2 had bulbar onset disease (A-B) and were age-matched to limb onset (A-L) [mean age 63.3 (SD ± 11.5) and 64.5 years (SD ± 11.6), respectively]. All participants donated blood samples at study inclusion (baseline). Seventeen pwALS (42.5%) were sampled at a second visit (V2) and 12 (30%) at a third visit (V3). As previously reported,^25^ the median time interval between V1 and V2 was 6.54 months [interquartile range (IQR) 4.83–8.28], with 7.21 months (IQR 5.31–9.26), between V2 and V3. Senescent T cell analysis was undertaken in a subset of pwALS [*n* = 31; mean age 64.7 (SD ± 8.7), 40% female] and NNC [*n* = 16; mean age 60.6 (SD ± 5.9), 35% female]. Evaluation of several key progression metrics including disease duration, ΔFRS, diagnostic delay, age at onset, sex and site of onset showed that A-F and A-S were more clearly separated using the latent class model compared to the ΔFRS approach (Supplementary Fig. 2). Accordingly, we took forward A-F and A-S classifications defined by the LCA approach for subsequent analyses.

**Table 1:** Demographic and clinical characteristics of the study cohorts under investigation

	Resolvin receptor analysis				SPM analysis plasma	
	T, B cells and monocytes		Senescent T cells			
	ALS	NNC	ALS	NNC	ALS	NNC
	*n* = 40	*n* = 20	*n* = 31	*n* = 16	*n* = 20	*n* = 10
Age, mean (±SD)	63.9 (±11.4)	60.4 (±7.1)	64.7 (±8.7)	60.6 (±5.9)	61.5 (±10.2)	61 (±11.4)
Gender (female %)	52.5	50	48.4	60	35	40
Site of disease onset (bulbar, %)	47.5	N/a	51.6	N/a	40	N/a
Disease onset—V1 (months), median (IQR)	16.5 (14.6)	N/a	15.7 (12.8)	N/a	13.9 (29.7)	N/a
ALSFRS-R at baseline, mean (±SD)	35.0 (±9.6)	N/a	36.6 (±9.3)	N/a	40.7 (±4.0)	N/a
ALSFRS-R change, mean (±SD)	0.87 (±0.6)	N/a	0.84 (±0.6)	N/a	N/a	N/a
Survival (months), median (IQR)	31.8 (21.1)	N/a	31.2 (17.2)	N/a	51.35 (58.7)	N/a

ALS, amyotrophic lateral sclerosis; IQR, interquartile range; N/a, not applicable; NNC, non-neurological control; SD, standard deviation; SPMs, specialized pro-resolving mediators; ALSFRS-R, ALS Functional Rating Scale Revised; ALSFRS-R change, the slope of the ALSFRS-R from baseline to the last visit divided by the time interval in months.

### SPM analysis

**Figure 1:**
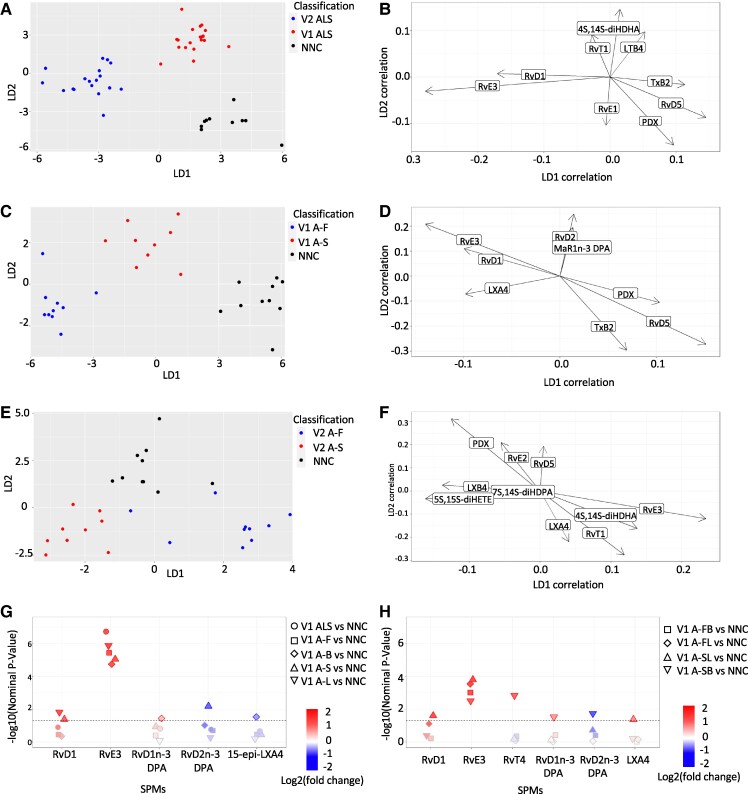
**LDA of lipid mediator plasma expression in pwALS and NNC.** LDA 2D score plots of lipid mediators (right side) representing comparisons among pwALS, ALS variants based on rate of disease progression (*n*: 20) and NNC (*n*: 10). Scatter plots (left side) of the topmost correlated lipid mediators to linear discriminant 1 (LD1) and linear discriminant 2 (LD2). (**A** and **B**) pwALS at baseline (baseline pwALS) versus pwALS at visit 2 (V2 pwALS) versus NNC at baseline. Baseline pwALS are represented by blue data points, V2 pwALS are represented by red data points and NNC are represented by black data points. (**C** and **D**) Fast-progressing pwALS at baseline (baseline A-F) versus slow-progressing pwALS at baseline (baseline A-S) versus NNC at baseline. Baseline A-F are represented by red data points, baseline A-S are represented by blue data points and NNC are represented by green data points. (**E** and **F**) Fast-progressing pwALS at Visit 2 (V2 A-F) versus slow-progressing pwALS at Visit 2 (V2 A-F) versus NNC at baseline. V2 A-F are represented by red, V2 A-S by blue and NNC are represented by green colour code. A-F, fast progressive ALS; A-S, slow progressive ALS; LDA, linear discriminant analysis; LD, linear discriminant; NNC, non-neurological controls; pwALS, patients with ALS; V2, Visit 2. (**G** and **H**) Manhattan plots representing differentially expressed resolvins. The horizontal line indicates the nominal *P*-value cut off of 0.05 (Wald test). Resolvins that are upregulated are highlighted with a red outline, whilst those that are downregulated are distinguished by a blue outline. A-F, fast progressive ALS (*n*: 20); A-FB, fast progressive ALS with bulbar onset (*n*: 10); A-FL, fast progressive ALS with limb onset (*n*: 10); A-S, slow progressive ALS; A-SB, slow progressive ALS with bulbar onset (*n*: 9); A-SL, slow progressive ALS with limb onset (*n*: 11); V2, Visit 2; NNC, non-neurological controls (*n*: 20); PwALS, participants with ALS.

SPMs including resolvins, maresins and protectins were measured at baseline and at the 12-month follow-up visit (Visit 2: V2) in blood samples from Cohort 1. Quality control checks of MS chromatograms were applied following published guidelines (29). Further review of SPM chromatograms revealed that of the total number of measurements (1800), 61% of baseline and V2 time/data points were below the quantification threshold (1100). The remaining 39% detectable SPMs (700) showed a median concentration of 0.76 pg/mL (IQR 0.37–2.19 pg/mL). For all time/data point measurements below the LOD, we utilized the minimum quantification level for the SPMs in the experiment. The percentage of time/data points below the LOD for each SPM species is reported in Supplementary Table 2. LDA showed segregation of ALS phenotypes from NNC along the LD dimensions, with resolvin concentrations in A-F, A-S and NNC performing best at baseline and at V2, and significantly changing between baseline and V2 in pwALS (Fig. 1A–F). PwALS at baseline had elevated blood expression of RvD1 and RvE3, along with decreased expression of RvD5, PDX and TxB2 compared to NNC. Notably, the A-F subgroup was further differentiated by a high expression of RvD2 and MaR1n-3 DPA, whilst LXA4 displayed a high blood expression in the A-S subgroup. Furthermore, at V2, pwALS had an increased expression of RvT1, LTB4 and 4s-14S-diHDHA, in addition to a decreased expression of RvD5, RvE2 and PDX. The A-F subgroup was further distinguished at V2 by a significantly high expression of LXB4, 5S-15S-diHETE and 7S-14S-diHDPA, whilst in the A-S subgroup at V2, there was an elevated expression of RvE3, RvT1, LXA4 and 4s-14S-diHDHA (Fig. 1A–F).

In agreement with the LDA approach, the differential expression analysis revealed a nominally significant increase in the expression of RvE3 in pwALS at baseline compared to NNC (nominal *P* = 1.91 × 10−07), an upregulation seen in all pwALS phenotypes (1.33 × 10−06 < nominal *P* < 0.00314). RvD1 displayed an upregulation in A-L, A-S and A-SL at baseline when compared to NNC (nominal *P* = 0.01523, 0.0416 and 0.0250, respectively). Additionally, RvD1n-3 DPA displayed an upregulation in A-B at baseline, particularly in A-SB, when compared to NNC (nominal *P* = 0.0367 and 0.0303). RvD2n-3 DPA exhibited a downregulation in A-S at baseline and Visit 2 (data not shown) (nominal *P* = 0.00659 and 0.0187, respectively) and in A-SB at baseline compared to NNC (nominal *P* = 0.0184). A-SB at baseline also exhibited an upregulation of RvT4 compared to NNC (nominal *P* =0.00147) (Fig. 1G and H).

In consideration of the reported SPM regulation in blood from pwALS involving RvD1, RvD1_n-3 DPA_, RvD2, RvD2_n-3 DPA_, RvD5, RvD6, RvE1, RvE2, RvE3, RvT1 and RvT4 mediators both at baseline and at V2, we proceeded with the investigation of the expression of resolvin-sensitive GPR32 and GPR18 receptors in subsets of blood mononuclear cells from a second cohort of pwALS.

### GPR32/GPR18 clustering and differential expression in PBMCs

Unbiased clustering of resolvin receptors GPR32 and GPR18 expressing cells including monocytes, B and T cell subtypes was performed using PhenoGraph. Data were also analysed using FlowSOM (Supplementary Table 3). The median GPR32 and GPR18 receptor expression was obtained by R-based differential comparison, using the diffcyt package,^39^ for each cell type. Below, we report data obtained from measurements of each mononuclear cell subtype undertaken at baseline.

#### Monocytes

**Figure 2:**
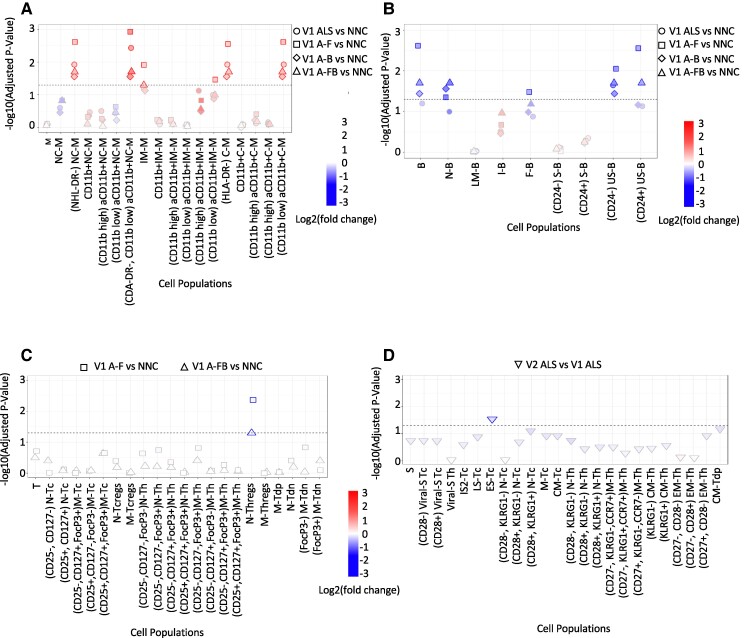
**GPR32 resolvin receptor median expression changes at baseline in mononuclear cell types from ALS phenotypic variants (*n*: 40) compared to NNC (*n*: 20) and pwALS at Visit 2 compared to Visit 1.** The horizontal line indicates the Wald test-adjusted (Benjamini–Hochberg) *P*-value cut off of 0.05. A change of colours from red to blue indicates a statistically significant upregulation of GPR32 versus a statistically significant downregulation of GPR32. (**A**) Monocytes, (**B**) B cells, (**C**) T cells and (**D**) senescent T cells. A-B, bulbar onset ALS (*n*: 19); A-F, fast-progressing ALS (*n*: 20); A-FB, fast-progressing ALS with bulbar onset (*n*: 10); A-S, slow-progressing ALS (*n*: 20); NNC, non-neurological controls; PwALS, participants with ALS; V2, Visit 2.

The 17 monocyte subtypes expressing GPR32 were found to be more significantly represented in pwALS, A-F, A-FB and A-B compared to NNC. Here, activated CD11b^+^ classical monocytes (HLA-DR-, CD11b Low) (*P* = 0.0024), activated CD11b^+^ non-classical monocytes (HLA-DR^−^, CD11b Low) (*P* = 0.0012), HLA-DR^−^ classical (*P* = 0.0027), intermediate (*P* = 0.0119) and non-classical monocytes (*P* = 0.0024) presented the highest GPR32 expression (Fig. 2A). There were no significant GPR18 expression changes in monocyte subtypes across ALS phenotypic variants compared to NNC (data not shown).

#### B cells

Eight B cell subtypes expressed both GPR32 and GPR18. At baseline and compared to NNC, GPR32 expression was reduced in B cell subtypes from A-F [total B cells *P* = 0.0023; unswitched memory B cells (CD24^+^) *P* = 0.0027; unswitched memory B cells (CD24^−^) *P* = 0.0087; follicular B cells *P* = 0.0327], from A-FB [total B cells *P* = 0.0195; unswitched memory B cells (CD24^+^) *P* = 0.0195; unswitched memory B cells (CD24^−^) *P* = 0.0195 and naïve B cells *P* = 0.0195) and from A-B (unswitched memory B cells *P* = 0.0361 and naïve B cells *P* = 0.0272) (Fig. 2B).

**Figure 3:**
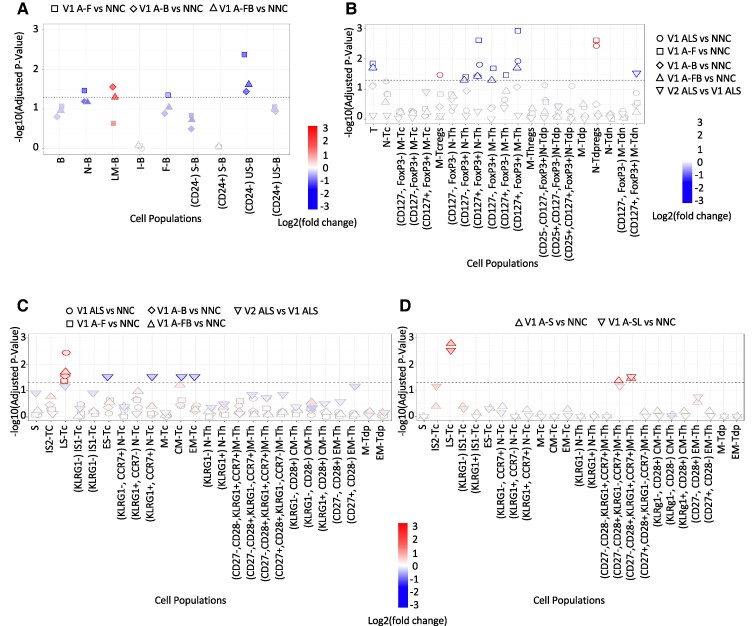
**GPR18 median expression changes at baseline in mononuclear cell types from ALS phenotypic variants (*n*: 40) compared to NNC (*n*: 20) and pwALS at Visit 2 compared to Visit 1.** The horizontal line indicates the Wald test-adjusted (Benjamini–Hochberg) *P*-value cut off of 0.05. A change of colours from red to blue indicates a statistically significant upregulation of GPR32 versus a statistically significant downregulation of GPR18. (**A**) B cells, (**B**) T cells and (**C** and **D**) senescent T cells. A-B, bulbar onset ALS (*n*: 19); A-F, fast-progressing ALS (*n*: 20); A-FB (*n*: 10), fast-progressing ALS with bulbar onset; A-S, slow progressive ALS (*n*: 20); AS-L (*n*: 10), slow-progressing ALS with limb onset; NNC, non-neurological controls (*n*: 20); PwALS, participants with ALS; V2, visit 2.

GPR18 median expression levels were also significantly lower in B cell subtypes from A-F [unswitched memory B cells (CD24^−^) *P* = 0.0041; naïve B cells *P* = 0.0337; follicular B cells *P* = 0.0439] and from A-FB [unswitched memory B cells (CD24^−^) *P* = 0.0237]. Only in late memory B cells from A-FB and from A-B were GPR18 expression levels found to be upregulated (*P* = 0.0496 and *P* = 0.0272, respectively) (Fig. 3A).

#### T cells

Twenty-one GPR32- and 20 GPR18-expressing T cell subsets were identified. GPR32 expression levels were significantly downregulated in naïve Tregs from A-F (*P* = 0.0042) and from A-FB (*P* = 0.049) compared to NNC (Fig. 2C). We also found a significantly reduced GPR18 expression on naïve T helper cells (CD25^−^, CD127^+^, FoxP3^+^) and memory CD127^−^ and CD127^+^ T helper cells (CD25^−^, FoxP3^+^) from pwALS (*P* = 0.0154 and *P* = 0.0117), A-F (*P* = 0.0023 and *P* = 0.0011) and A-FB (*P* = 0.0367 and *P* = 0.0195) compared to NNC (Fig. 3B). In A-F, GPR18 expression was significantly reduced on T cells (*P* = 0.0138), naïve T helper cells (CD25^−^, CD127^−^, FoxP3^+^) (*P* = 0.0382), and CD127^+^ and CD127^−^ memory T helper cells (CD25^−^, FoxP3^−^) (*P* = 0.0337 and 0.0201) (Fig. 3B). In contrast, GPR18 was upregulated in naïve double positive Tregs (*P* = 0.0023) from A-F compared to NNC. Additionally, upregulated GPR18 expression levels were observed in pwALS on naïve double positive Tregs (*P* = 0.0037) and memory cytotoxic (CD8^+^) Tregs (*P* = 0.0338) (Fig. 3B).

#### Senescent T cells

Twenty-three GPR32 and 24 GPR18 clusters expressing senescent T cell subsets were identified. There were no significant differences in lymphocyte GPR32 expression between ALS clinical phenotypes and NNC. In contrast, GPR18 was significantly upregulated in late senescent cytotoxic (CD8^+^) T cells from pwALS (*P* = 0.0037), A-F (*P* = 0.0431), A-B (*P* = 0.0272), A-FB (*P* = 0.0195), A-S (*P* = 0.0016) and A-S with limb onset (A-SL) (*P* = 0.0029) compared to NNC (Fig. 3C and D). Upregulation of GPR18 expression was also seen on M-Th (CD27^−^, CD28^+^, KLRG1^−^, CCR7^+^) from A-S (*P* = 0.0342) compared to NNC (Fig. 3D). M-Th (CD27^−^, CD28^+^, KLRG1^+^, CCR7^+^) were increased from A-S (*P* = 0.0423) and from A-S with limb onset (*P* = 0.0295) compared to NNC (Fig. 3D).

### Longitudinal analysis

Changes in the expression of GPR32 and GPR18 at V2 and V3 compared to baseline levels were calculated using R-based differential comparisons for each cell type under investigation. GPR32-expressing early senescent cytotoxic (CD8^+^) T cells were significantly downregulated in V2 compared to baseline (*P* = 0.0291) (Fig. 2D). Similarly, GPR18 expression was reduced in central memory cytotoxic T cells (*P* = 0.0291), effector memory cytotoxic T cells (CD27^+^, CD28^−^, KLRG1^+^) (*P* = 0.0291) and early senescent cytotoxic (CD8^+^) T cells (*P* = 0.0291) in V2 compared to baseline (Fig. 3C). Frequencies of GPR18 were significantly downregulated in memory double negative T cells (CD4^−^, CD8^−^, CD25^−^, CD127^+^, FoxP3^+^) in V2 pwALS compared to baseline (Fig. 3B).

### Survival analysis

**Figure 4:**
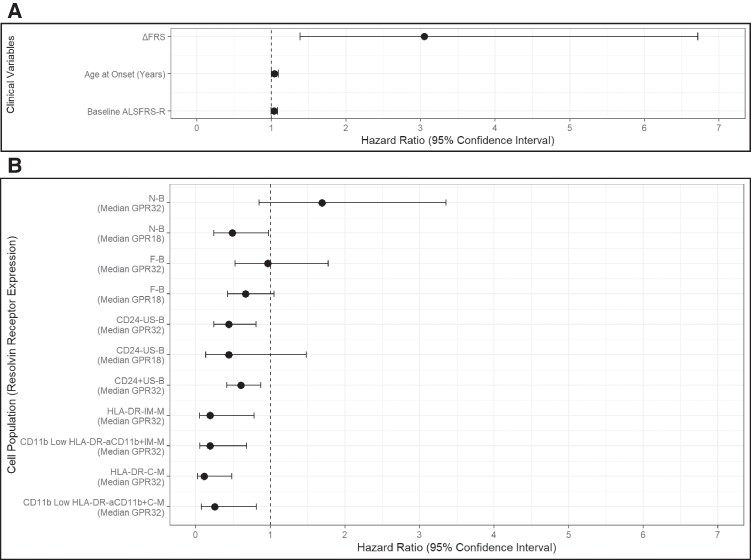
**Forest plots depicting Cox proportional hazards models’ variables. A** illustrates the Cox proportional hazards model derived exclusively from clinical variables. **B** displays HRs for the cell-specific GPR32 and GPR18 median expression values from each Cox proportional hazards model incorporating GPR32 or GPR18 median expression values in the named cell type alongside variables from the clinical model. Cell types were obtained through PhenoGraph clustering. The HR is represented by a solid black circle, with the 95% CI indicated by the horizontal line. The dashed vertical line signifies a HR of 1. HRs below 1 are associated with longer survival, whilst HRs above 1 are associated with shorter survival. Population in study: pwALS *n*: 40; NNC *n*: 20. ΔFRS: the difference of ALSFRS-R approximated to 48 at disease onset (neurologically healthy) and of ALSFRS-R at baseline sampling divided by the time interval in months. ALSFRS-R, ALS Functional Rating Scale Revised.

The correlation between survival and clinical and biological variables was examined using Cox proportional hazards analysis. Using a stepwise approach, we identified the model with the best combination of clinical and demographic predictors, which included age at onset and ΔFRS. In line with previous reports,^51^ older age at onset [hazard ratio (HR): 1.04, 95% confidence interval (CI): 1.09, 1.00; *P* = 0.05] and higher ΔFRS (HR: 3.05, 95% CI: 6.72, 1.38; *P* = 0.01) predicted shorter survival (Fig. 4A; Supplementary Table 4). Higher ALSFRS-R at baseline did not reach statistical significance (HR: 1.04, 95% CI: 1.08, 0.997; *P* = 0.07).

This model was then extended and compared to further Cox models that included GPR32 and GPR18 resolvin receptor median expression levels in each of the cell subtypes under investigation alongside the clinical variables. Separate models were created for each cell type and resolvin receptor expression level. ANOVA determined that the inclusion of most cell types’ resolvin receptor expression significantly improved model fit (Table 2). We tested Cox models primarily based on data from PhenoGraph and validated the results using data from FlowSOM as a sensitivity analysis. Significant associations with longer survival were observed for higher GPR32 median expression on CD24^+^ and CD24^−^ unswitched memory B cells (HR: 0.604, 95% CI: 0.873, 0.418; *P* = 0.007 and HR: 0.444; 95% CI: 0.806, 0.245; *P* = 0.008, respectively), classical and intermediate monocytes (HLA-DR^−^) (HR: 0.112, 95% CI: 0.486, 0.026; *P* = 0.003 and HR: 0.192, 95% CI: 0.782, 0.047; *P* = 0.021, respectively) and activated CD11b^+^ classical and intermediate monocytes (CD11b Low HLA-DR^−^) (HR: 0.251, 95% CI: 0.815, 0.077; *P* = 0.021 and HR: 0.193, 95% CI: 0.681, 0.055; *P* = 0.011, respectively) (Fig. 4B and Table 3). Consistent with the GPR32 findings, higher GPR18 median expression on CD24^−^ unswitched memory B cells (HR: 0.441, 95% CI: 1.48, 0.131; *P* = 0.186) and naïve B cells (HR: 0.49, 95% CI: 0.976, 0.246; *P* = 0.043) were associated with longer survival (Fig. 4B). The only finding that was not replicated using both clustering algorithms was the association between GPR32 median expression in CD24^+^ unswitched memory B cells, which was significant using PhenoGraph but not using FlowSOM data.

**Table 2:** Comparison of Cox proportional hazards models for survival from the onset of ALS, incorporating clinical and biological factors versus a model based solely on clinical factors

Model	Log likelihood	*χ* ^2^	Degrees of freedom	*P*-value
Clinical	−46.368			
N-B (median GPR32)	−44.749	3.237	1	0.072
N-B (median GPR18)	−43.199	6.337	1	**0**.**012**
F-B (median GPR32)	−46.363	0.009	1	0.925
F-B (median GPR18)	−45.041	2.653	1	0.103
CD24-US-B (median GPR32)	−42.547	7.643	1	**0**.**006**
CD24-US-B (median GPR18)	−44.204	4.328	1	**0**.**037**
CD24^+^ US-B (median GPR32)	−44.268	4.199	1	**0**.**040**
HLA-DR-IM-M (median GPR32)	−43.621	5.494	1	**0**.**019**
CD11b Low HLA-DR-aCD11b^+^IM-M (median GPR32)	−41.763	9.209	1	**0**.**002**
HLA-DR-C-M (median GPR32)	−41.629	9.478	1	**0**.**002**
CD11b Low HLA-DR-aCD11b^+^C-M (median GPR32)	−44.393	3.949	1	**0**.**047**

Each model includes the pre-defined predictors used in the clinical model and the median resolvin receptor expression for a given cell type, determined through unbiased clustering using PhenoGraph. Significant *P*-values (<0.05; Wald test) are indicated in bold.

**Table 3:** Summary of Cox proportional hazards models for survival, incorporating clinical variables and cell subtype-specific median expression levels of GPR32 and GPR18

Variable	Regression coefficient	Regression coefficient robust standard error	Hazard ratio (95% confidence interval)	*P*-value
N-B (median GPR32)	0.525	0.349	1.69 (3.35, 0.852)	0.133
N-B (median GPR18)	−0.714	0.352	0.49 (0.976, 0.246)	**0**.**043**
F-B (median GPR32)	−0.033	0.310	0.967 (1.78, 0.527)	0.914
F-B (median GPR18)	−0.405	0.231	0.667 (1.05, 0.424)	0.080
CD24-US-B (median GPR32)	−0.811	0.304	0.444 (0.806, 0.245)	**0**.**008**
CD24-US-B (median GPR18)	−0.818	0.618	0.441 (1.48, 0.131)	0.186
CD24^+^US-B (median GPR32)	−0.505	0.188	0.604 (0.873, 0.418)	**0**.**007**
HLA-DR-IM-M (median GPR32)	−1.650	0.716	0.192 (0.782, 0.0472)	**0**.**021**
CD11b Low HLA-DR-aCD11b^+^IM-M (median GPR32)	−1.644	0.643	0.193 (0.681, 0.0548)	**0**.**011**
HLA-DR-C-M (median GPR32)	−2.192	0.750	0.112 (0.486, 0.0257)	**0**.**003**
CD11b Low HLA-DR-aCD11b^+^C-M (median GPR32)	−1.382	0.601	0.251 (0.815, 0.0774)	**0**.**021**

Cell subtypes were determined using the PhenoGraph clustering algorithm. Significant *P*-values (<0.05; Wald test) are indicated in bold. A negative regression coefficient indicates an association with longer survival.

Using correlation analysis, we identified two distinct groups of cell types exhibiting highly correlated median resolvin receptor expression. The first group comprised of various subtypes of monocyte cells, whilst the second group consisted of different subtypes of B cells (Supplementary Fig. 5). This finding underscores a strong relationship between resolvin receptor expression levels within each cell type, emphasizing their unique associations. Given the high correlation among our biological markers, we refrained from adjusting the *P*-values in our survival analysis to prevent an inflated risk of false negatives. This decision was made because correlated variables are likely influenced by similar underlying factors, violating the assumption of independence necessary for *P*-value adjustment.

## Discussion

We report the regulation of endogenous molecular pathways involved in the resolution of inflammation, including higher concentrations of resolvin lipid mediators in blood from pwALS with a slower functional decline, and the differential expression of resolvin receptors on cell membrane of blood monocyte and lymphocyte subpopulations from pwALS with a faster disease progression. Our clustering analysis of blood mononuclear cells indicates that a range of monocyte and lymphocyte subpopulations previously implicated in the systemic immune dysregulation seen in ALS express both GPR18 and GPR32 resolvin receptors. GPR32 receptor expression is increased in circulating monocytes with a known anti-inflammatory phenotype whilst GPR18 receptor is upregulated in lymphocytes with a recognized senescent phenotype. We have also identified lower expression levels of GPR32 and of GPR18 receptors in several lymphocyte subsets from pwALS with a more aggressive disease phenotype. Prognostically unfavourable clinical features in more severely affected pwALS include a more rapid disease development to nutritional and/or respiratory failure and bulbar onset of disease, which is often seen in association with early signs of frontotemporal involvement.^52^ In our multivariate survival analysis, the inclusion of high expression levels of resolvin receptors in blood mononuclear cells, along with pre-defined clinical variables, significantly improves the prediction of survival compared to a model that relies solely on clinical variables. Notably, high expression of GPR32 and GPR18 in circulating B and myeloid cells is associated with increased survival. These data support but do not confirm a link between higher circulating resolvin mediators and a more benign disease phenotype. The activation of SPM receptor pathways may attenuate inflammation, improve neuronal resilience and reduce the pace of disease progression in pwALS. Under this assumption, the over-expression of SPM target cell receptors may be an adaptive response to the lack or insufficient activation of resolvin-mediated rescuing pathways. Whilst our data are preliminary and require validation in much larger ALS cohorts, a resolvin-mediated therapeutic approach to attenuate or arrest inflammation and to improve clinical outcomes should be considered in the subset of pwALS with a more aggressive form of the disease.

Our MS-based lipid mediator analysis reveals that among detectable SPMs, higher blood levels of resolvins identify pwALS with a slow progressing disease (A-S) (Fig. 1). This observation aligns to experimental evidence that has implicated lipid metabolism to the unravelling of neurodegeneration and ultimately to disease progression. Changes in the metabolism of lipids such as cholesterol and phospholipids and their accumulation within neurons and glial cells are thought to be linked to regulation of neuroinflammation. Among those lipids with a regulatory function on inflammation, resolvins are among endogenous molecules able to mitigate chronic inflammatory responses, normally associated with a compromised immunological tolerance. Tregs are also critical to immunological homeostasis. Tregs frequencies in relation to CD4 lineages are known to be reduced in ALS, and the downregulation of these immune regulators is inversely correlated with the rate of disease progression and directly to length of survival.^53^ RvD1 has been shown to increase anti-inflammatory Tregs *in vitro* and to correct the Treg/Th17 imbalance seen in conditions like lupus erythematosus, reducing the differentiation of these cells into a pro-inflammatory (Th17) phenotype, known to produce IL-17, IL-22 and IL-23 and to recruit neutrophils to sites of inflammation.^54,55^

Resolvin-mediated modulation of inflammatory occurs via G protein-coupled receptors expressed on leucocytes and myeloid cells. For example, RvD1’s effect is mediated by GPR32 and FPR2/ALX receptors. RvD1 is a potent ligand showing effects at nanogram levels,^6^ and recent studies have suggested that the effect of RvD1 is strictly GPR32 receptor dependent.^56^ Changes in GPR32 expression on cells is therefore bound to reflect changing concentrations of circulating RvD1. Our study shows measurable RvD1 concentrations in blood from pwALS in the picomolar range (median 0.47 pg/mL; 0.06–14.69; Supplementary Table 2). Sixty-one per cent of data/time points in the study were below the lower LOD, and slow ALS progressors showed the highest RvD1 detectable concentrations in blood. Whilst low blood concentrations of these lipid mediators may put into question a role for resolvins in inflammation reduction, the higher concentrations of resolvins seen in slower progressing ALS support a role for these mediators in the modification of those inflammatory responses linked to disease progression.

We have also shown that RvD1 receptor GPR32 is highly expressed in circulating monocytes from fast-progressing and bulbar onset pwALS, the subset of pwALS with the worse disease prognosis and low RvD1 blood concentration. Among these monocytes, the subset of myeloid cells expressing the inflammation-suppressing CD11b^+^ integrin has the highest GPR32 expression (*P* < 0.0024). We have previously shown that higher blood frequencies of CD11b^+^ monocytes are associated with slower progression of the disease.^5^ Treatment with RvD1 may therefore have the potential of altering the balance of myeloid cells in circulation through a GPR32 receptor-mediated anti-inflammatory effect and via enhancement of CD11b^+^ monocytes. However, the concentrations at which RvD1 in blood may have true biological effects on these macrophage precursors are unknown. Further studies into the stereochemical mechanisms and the potency of these bioactive mediators using synthetic compounds to be tested in *in vitro* and animal models of ALS are needed.

The activation of SPM receptors is thought to result in efficient resolution of inflammation via apoptosis, phagocytosis and efferocytosis, involving mostly polymorphonuclear leucocytes (neutrophils) and macrophages. RvD2 interacts with GPR18, which is expressed in polymorphonuclear neutrophils, monocytes and macrophages.^57^ RvD2-mediated activation of GPR18 is known to orchestrate the resolution of acute inflammation, by limiting polymorphonuclear neutrophils infiltration and enhancing phagocyte antigen clearance. Our mononuclear cell clustering and expression analysis of GPR32 and GPR18 showed that these receptors were minimally or not expressed in most B cell subsets, naïve helper T cells and Tregs in pwALS with faster progressing and bulbar onset disease, the same clinical variant of ALS individuals with a low concentration of resolvins in circulation. In contrast, GPR18 was upregulated in naïve double positive Tregs, memory cytotoxic Tregs, senescent late memory B cells and late senescent CD8^+^ T cells from most ALS phenotype variants compared to NNC (*P* < 0.0431). It has been proposed that sustained antigenic stimulation throughout life facilitates the development of senescence in T and B cells, for example switching CD8^+^ T and B cells towards a senescent and memory phenotype, contributing to the loss of immune tolerance and to the low-grade systemic inflammation that is seen in age-related diseases. CD8^+^ senescent T and late memory B cells are highly inflammatory, secrete cytotoxic mediators and express natural killer cell receptor.^58,59^ Higher frequencies of CD8^+^ senescent T and late memory B cells, along with long-term activation of myeloid cells, are often seen in the context of a low-grade systemic inflammation, a key pathophysiological component of atherosclerotic cardiovascular disease, obesity and of neurodegenerative disorders.^60,61^ Our study confirms the upregulation of GPR18 in senescent T and B cells from pwALS and specifically in fast-progressing and bulbar onset patients. It may therefore be possible to exploit the inflammation resolution effect of RvD2, or of other GPR18-sensitive ligands, to mitigate the known harmful effect of these senescent cells.

In support of our finding of a downregulation of resolvin receptors in mononuclear cells in pwALS with a more aggressive phenotype, our multivariate survival analysis shows a strong positive effect on survival of high expression levels of GPR32 and GPR18 receptors in blood mononuclear cells. When considering the contribution of each individual GPR32/GPR18-expressing cell type, we observe that the predictors with the most significant effect are resolvin receptor expression on classical monocytes (HR: 0.11, *P* = 0.003) and unswitched memory B cells (HR: 0.44, *P* = 0.008). This observation highlights the role of circulating B cells and monocytes in the pathogenesis of ALS, a promising area of investigation into the disease immunopathology.^62^ It is not clear what role unswitched memory B cells and late memory B cells, the latter among the most pro-inflammatory B cell subsets, may have in the immune dysregulation reported in ALS^63^ and how any treatment based on increasing endogenous resolvin concentration may affect the relative balance of these cells’ phenotype.

Our study has limitations in so far the pwALS subgroups chosen to enable the study of SPM pathway may not be fully representative of the ALS population. The lack of a complete understanding of the role that the SPM pathway has in inflammation control may also limit our interpretation of the results. Firstly, as our main research focus is the regulation of SPMs and of their receptors in relation to rate of disease progression, we have selected cohorts with an equal distribution of ALS patients for gender and site of onset (bulbar versus limb) (Table 1). Whilst this is justified by the need to reduce any gender and site of onset-specific effect on the development of neuroinflammation, it is important to stress that male and limb onset cases are normally the majority in the ALS population. Furthermore, selection of pwALS in Cohort 1 was done to include a proportion of atypically slow progressing ALS to test the hypothesis that a more indolent ALS phenotype is associated to higher expression of SPMs involved in resolution of inflammation [baseline ALSFRS-R mean (±SD) 40.7 (±4.0); survival median (IQR) 51.35 (58.7), Cohort 1; Table 1]. Whilst unsupervised data processing is applied to adjust for clinical variability, the inclusion of a significant proportion of pwALS with slow disease progression makes our data less generalizable to the overall ALS population, where a more aggressive disease course is often seen. Future studies will have to include larger samples of ALS cases more representative of the ALS clinical heterogeneity, adjusting for demographic and clinical composition of ALS in the data analysis. The incomplete understanding of the biology of lipid mediators of inflammation resolution and of the effects of ligand–receptor interactions on immune cells makes data interpretation difficult. The major limitation in our investigation and of several studies that have addressed neuroinflammation through the lens of systemic changes is that there has been little progress towards linking the profile of peripheral alteration to the overall dysregulation of the immunological system that affects motor cells in spinal cord and brain. However, separate investigations have revealed post-mortem ALS spinal cord infiltration by macrophages and T cells, including caspase-positive neurons, IL-6- and TNF-α-positive macrophages.^16^ Aggregated superoxide dismutase-1 treatment of these macrophages led to the activation of cyclooxy-genase-2 and caspase-1 and to the expression of the inflammatory cytokines IL-1β, IL-6 and TNF-α, a process that could be inhibited using lipid mediator resolvin D1 (RvD1). These findings along with data obtained from animal models where both fluids and affected tissues are readily available provide indications that changes occurring in the periphery may represent a readout and reflect immunological alterations within affected tissues surrounding motor cells.

Our findings may also indicate an altered ligand–receptor feedback (e.g. RvD1 and GPR32) leading to a disruption of the resolution of inflammation and to a faster pace of neuronal degeneration. We do not fully understand the potency and cell specificity of SPMs (or of their synthetic substitutes) in relation to blood concentrations. Endogenous resolvin anti-inflammatory effects have also been called into question due to their picogramme-range low abundance in blood. In addition, mononuclear cells may become activated by multiple ligands in what is described as receptor pleiotropy,^64^ making it difficult to disentangle the real resolving-mediated effect. It is, nevertheless, accepted that synthetic resolvins can be used successfully to modulate the inflammatory response, including in neurological conditions as demonstrated in several experimental animal models and in the first clinical trials in humans.^65^ We can speculate that a resolvin-based therapeutic strategy will have to be a cell selective treatment inhibiting only those cells that are known to be toxic or enhancing those that positively impact immune tolerance like Tregs. To develop a therapy that reduces inflammation, we will have to also understand the biological rationale of the observed increase of RvD1, RvE3 and RvT4 concentrations in slow progressing ALS, and the relatively low RvD1-sensitive GPR32-expressing B and T cell frequencies in pwALS in our ALS cohorts. From a therapeutic perspective, RvD1-mediated effect may counter inflammation by correcting the Tregs/Tregs 17 imbalance that is specific to fast-progressing pwALS. A similar treatment approach based on Tregs enhancement, via low-dose IL-2, has recently shown promising results in a large, multi-centre, placebo-controlled clinical trial in ALS.^66^

Our study points to the potential of a targeted approach to attenuate the toxic effect that immune dysregulation and high levels of senescent lymphocytes may have in the progression of ALS. Further natural history studies interrogating the systemic inflammatory response and endogenous mechanisms of resolution of inflammation in ALS progression are needed to pave the way to novel therapeutics for ALS, a neurodegenerative condition with an increasingly recognized role of neuroinflammation in the progression of the disease.

## Supplementary Material

fcae402_Supplementary_Data

## Data Availability

The data sets used in the work described in this article are available from the corresponding author on reasonable request. The codes created for machine learning analysis in this study are deposited in the GitHub repository: https://github.com/guypwhunt/resolvinAndResolvinReceptorAnalysis. Biological samples and data used in the investigation have been obtained from ALS Biomarkers Study (East London Research Ethics Committee, London, UK—REC reference 09/H0703/27) and for A Multicentre Biomarker Resource Strategy In ALS (‘AMBRoSIA’, South-East Research Ethics Committee—16/LO/2136).
