# Supplementary material for: Lipid-mediated resolution of inflammation and survival in amyotrophic lateral sclerosis
Source: Brain Commun. 2025 Jan 15;7(1):fcae402. doi: 10.1093/braincomms/fcae402 (PMC11733686; doi:10.1093/braincomms/fcae402)
Supplement: fcae402_Supplementary_Data [file fcae402_supplementary_data.docx]

**Supplementary Data**

**Supplementary Figures**


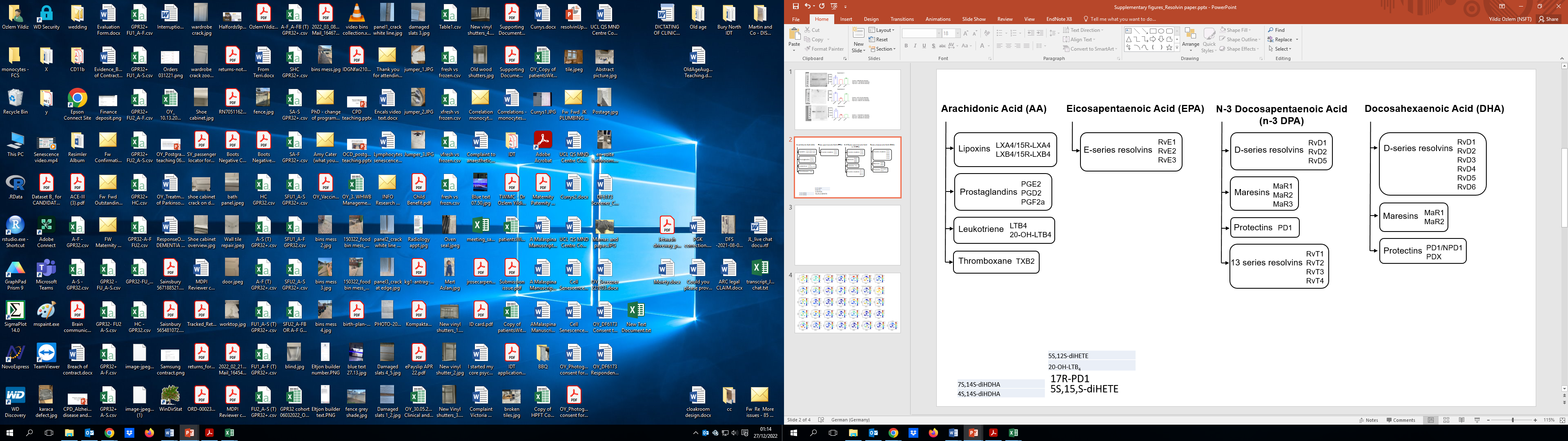


**Supplementary Figure 1: The four major fatty acid metabolomes and lipid mediators under investigation.**


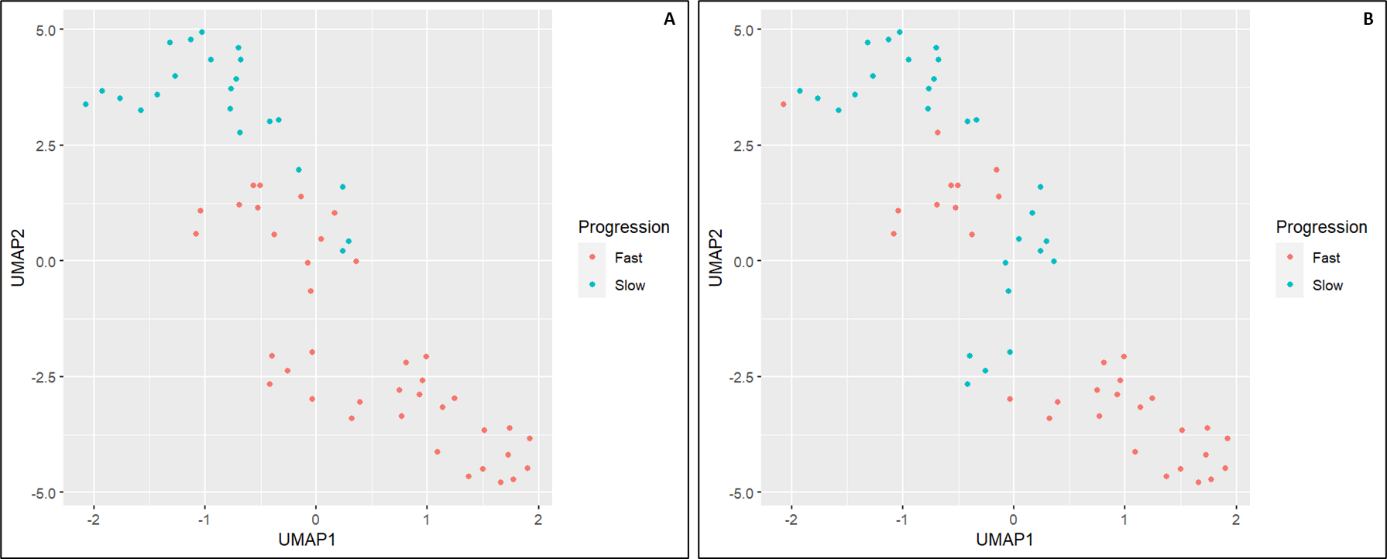


**Supplementary Figure 2: UMAP plots representing ALS disease progression defined by (A) latent class model and (B) standard ΔFRS approach.** Fast progressing ALS (A-F; n: 20) was defined as ΔFRS >0.5 points / month and slow progressing ALS (A-S; n: 20) as ΔFRS <0.5 points / month. UMAP was performed on uncensored ALS patients’ disease duration, ΔFRS, diagnostic delay, age at onset, sex, and site of onset. UMAP1 exhibits the highest correlation with ΔFRS (correlation coefficient = 0.805), while UMAP2 shows the strongest correlation with both disease duration (correlation coefficient = 0.761) and diagnostic delay (correlation coefficient = 0.753).


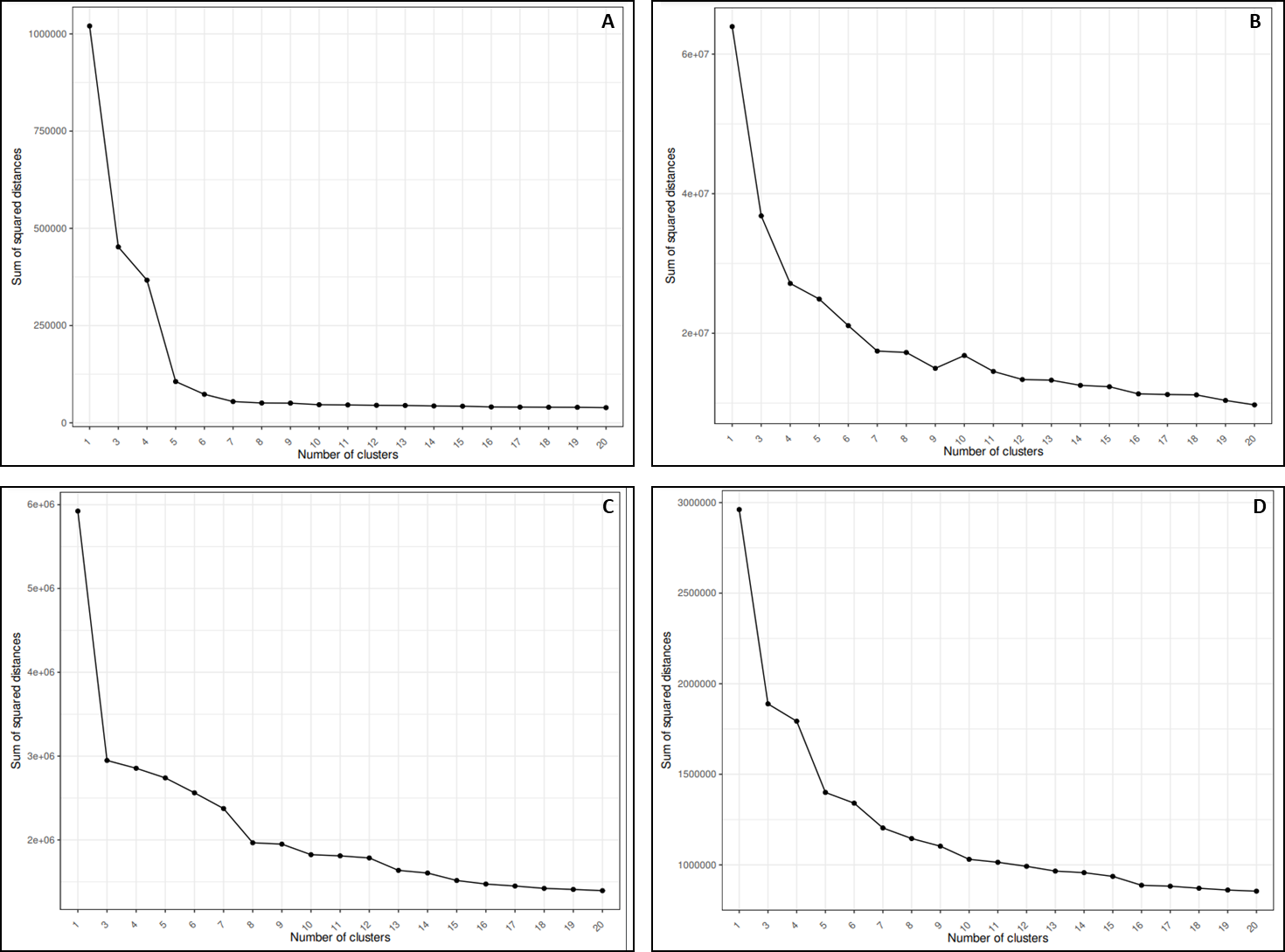


**Supplementary Figure 3: FlowSOM Elbow Plots for (A) B Cells, (B) Monocytes, (C) T Cells, and (D) senescent T Cells.**


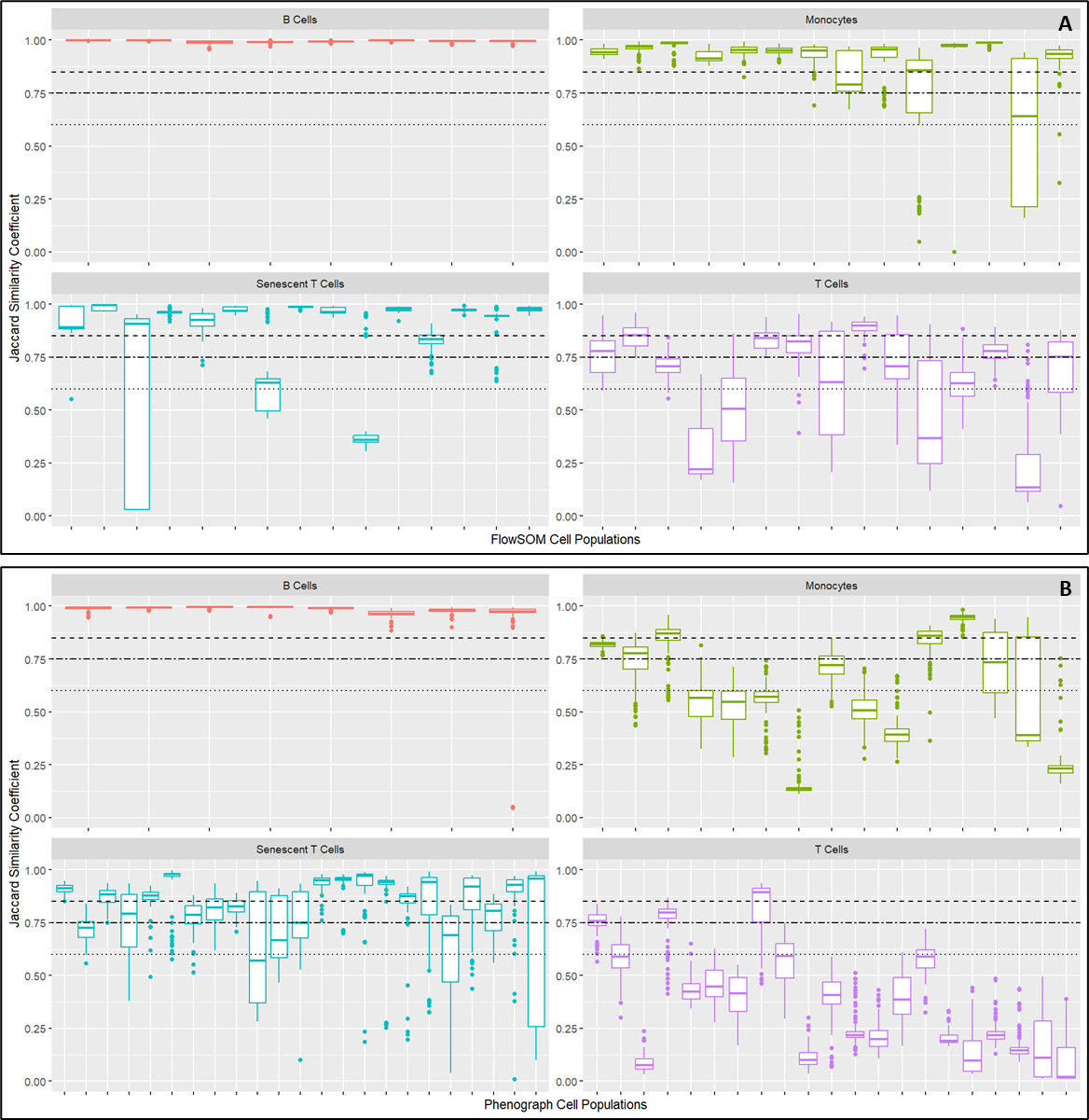


**Supplementary Figure 4: Boxplots of the Jaccard Similarity Coefficient for cell populations derived from (A) FlowSOM and (B) PhenoGraph.** The horizontal lines indicate the cut-off points for cell population stability categorisation. Median Jaccard similarity coefficient: >0.85 = very high stability, 0.85-0.75 = high stability, 0.75-0.6 = moderate stability, and <0.6 = low stability. Population in study: pwALS n: 40; NCC: 20.


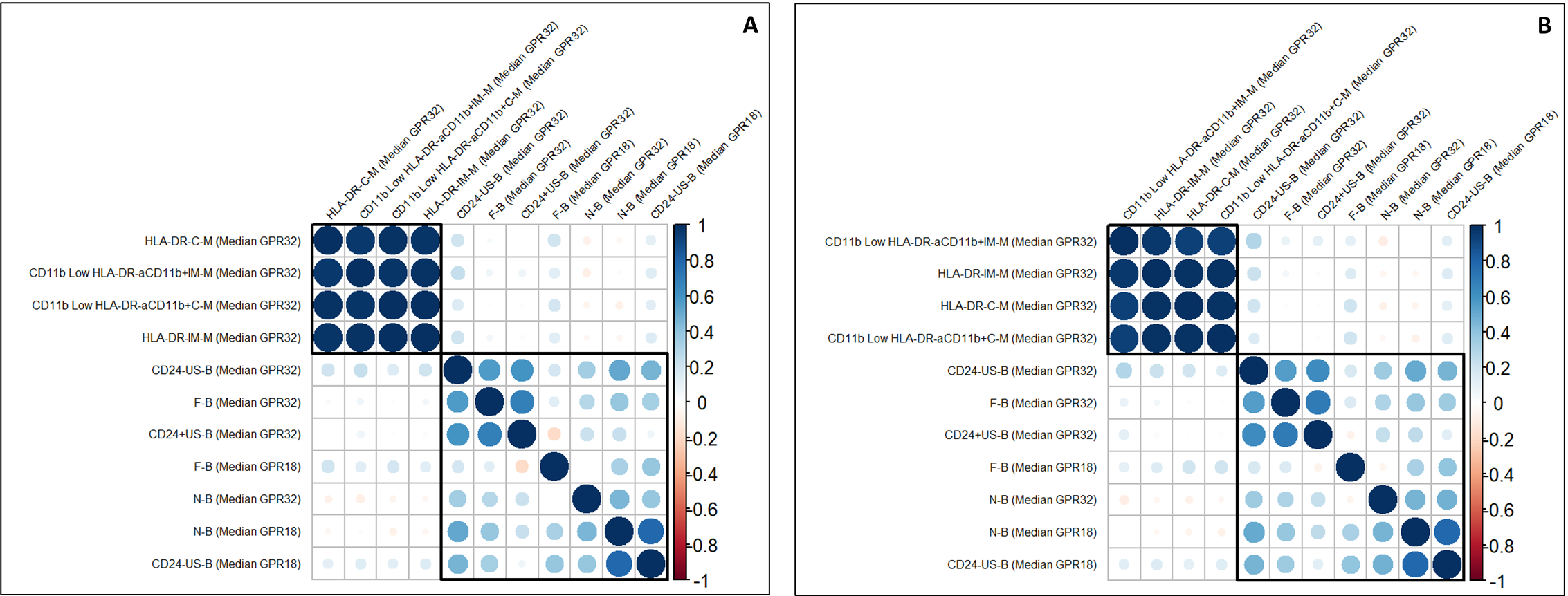


**Supplementary Figure 5: Matrix plots depicting the correlations between median GPR32 and GPR18 expression in cell populations derived from (A) FlowSOM and (B) PhenoGraph.** Black rectangles denote hierarchical clusters identified using the ward method. Positive correlations are visualized in blue, while a correlation of 0 is indicated in white and negative correlations are represented in red. Population in study: pwALS n: 40; NCC: 20.

**Supplementary Tables**

| **FC - MARKERS** | **SUBSETS** | **ABBREVIATION** |
| --- | --- | --- |
| **B CELL MARKERS** | | |
| CD19+ (% lymphocytes) | All B cells | B cells |
| CD19+CD27- (% B) | Naïve B cells | N-B |
| CD19+CD27+ (% B) | Memory B cells | M-B |
| CD19+CD27-CD24+IgD- (% CD24+ N-B) | Immature B cells | I-B |
| CD19+CD27-CD24+IgD+ (% CD24+ N-B) | Follicular B cells | F-B |
| CD19+CD27-CD24+IgDlow  (% CD24+ N-B) | Transitional B cells | T-B |
| CD19+CD27+IgD- (% B) | Switched B cells | S-B |
| CD19+CD27+IgD+ (% B) | Unswitched B cells | US-B |
| CD19+CD27-IgD- (% B) | Late memory B cells | LM-B |
|  |  |  |
| **MONOCYTE MARKERS** |  |  |
| % monocytes | All monocytes | M |
| HLA-DR+CD14-CD16+ (%monocytes) | Non-classical monocytes | NC-M |
| HLA-DR+CD14+CD16+ (%monocytes) | Intermediate monocytes | IM-M |
| HLA-DR+CD14+CD16- (%monocytes) | Classical monocytes | C-M |
| CD11b+HLA-DR+CD14-CD16+ (%NC-M) | CD11b+ Non-classical monocytes | CD11b+NC-M |
| CD11b+HLA-DR+CD14+CD16+ (%IM-M) | CD11b+ Intermediate monocytes | CD11b+IM-M |
| CD11b+HLA-DR+CD14+CD16- (%C-M) | CD11b+ Classical monocytes | CD11b+C-M |
| Active CD11b+HLA-DR+CD14-CD16+ (%NC-M) | Active CD11b+ Non-classical monocytes | aCD11b+NC-M |
| Active CD11b+HLA-DR+CD14+CD16+ (%IM-M) | Active CD11b+ Intermediate monocytes | aCD11b+IM-M |
| Active CD11b+HLA-DR+CD14+CD16- (%C-M) | Active CD11b+ Classical monocytes | aCD11b+C-M |
|  |  |  |
| **T CELL MARKERS** |  |  |
| CD3+ (% lymphocytes) | All T cells | T cells |
| CD4+ (% T) | T helper cells | Th |
| CD8+ (% T) | CD8+ T cells | Tc |
| CD4+CD45RO+ (% CD4) | Memory T helper cells | M-Th |
| CD4+CD45RO- (% CD4) | Naïve T helper cells | N-Th |
| CD8+CD45RO+ (% CD8) | Memory CD8+ T cells | M-Tc |
| CD8+CD45RO- (% CD8) | Naïve CD8+ T cells | N-Tc |
| CD4+CD25high+CD127low (% CD4) | T regulatory cells | Tregs |
| CD4+CD25high+CD127low+FoxP3+ (% CD4) | FoxP3+ T regulatory cells | FoxP3+ Tregs |
| CD25high+CD127low+FoxP3+45RO+ (% CD4) | Memory T regulatory cells | M-Tregs |
| CD25high+CD127low+FoxP3+45RO- (% CD4) | Naïve T regulatory cells | N-Tregs |
| CD4+CD45RO+CD25high+CD127low+FoxP3+45RO+ (% CD4) | Memory T helper regulatory cells | M-Thregs |
| CD4+CD45RO-CD25high+CD127low+FoxP3+45RO+ (% CD4) | Naïve T helper regulatory cells | N-Thregs |
| CD8+CD45RO+CD25high+CD127low+FoxP3+45RO+ (% CD8) | Memory cytotoxic T regulatory cells | M-Tcregs |
| CD8+CD45RO-CD25high+CD127low+FoxP3+45RO+ (% CD8) | Naïve cytotoxic T regulatory cells | N-Tcregs |
| CD8+CD4+CD45RO+ (% T) | Memory double positive T cells | M-Tdp |
| CD8+CD4+CD45RO- (% T) | Naïve double positive T cells | N-Tdp |
| CD8-CD4-CD45RO+ (% T) | Memory double negative T cells | M-Tdn |
| CD8-CD4-CD45RO- (% T) | Naïve double negative T cells | N-Tdn |
|  |  |  |
| **T CELL SENESCENCE MARKERS** | | |
| CD8+ (% lymphocytes) | Cytotoxic T cells | Tc |
| CD8+CD45RA+CCR7-CD28+CD27+  (% CD8) | Early senescent cytotoxic T cells | ES-Tc |
| CD8+CD45RA+CCR7-CD28-CD27+  (% CD8) | Intermediate senescent cytotoxic T cells 1 | IS1-Tc |
| CD8+CD45RA+CCR7-CD28-CD27-  (% CD8) | Late senescent cytotoxic T cells | LS-Tc |
| CD8+CD45RA+CCR7-CD28+CD27-  (% CD8) | Intermediate senescent cytotoxic T cells 2 | IS2-Tc |
| CD8+CD27-CD45RA+KLRG1- (% CD8) | Non-viral associated senescent cytotoxic T cells | Non-viral-S Tc |
| CD8+CD27-CD45RA+KLRG1+(% CD8) | Viral associated senescent cytotoxic T cells | Viral-S Tc |
| CD4+ (% lymphocytes) | T helper cells | Th |
| CD4+CD45RA+CCR7-CD28+CD27+ (%CD4) | Early senescent T helper cells | ES-Th |
| CD4+CD45RA+CCR7-CD28+CD27- (%CD4) | Intermediate senescent T helper cells 1 | IS1-Th |
| CD4+CD45RA+CCR7-CD28-CD27- (%CD4) | Late senescent T helper cells | LS-Th |
| CD4+CD45RA+CCR7-CD28-CD27+ (%CD4) | Intermediate senescent T helper cells 2 | IS2-Th |
| CD4+CD27-CD45RA+KLRG1- (%CD4) | Non-viral associated senescent T helper cells | Non-viral-S Th |
| CD4+CD27-CD45RA+KLRG1+ (%CD4) | Viral associated senescent T helper cells | Viral-S Th |

**Supplementary Table 1. List of flow cytometry (FC) markers for B cells, Monocytes, T cells including senescent T cells.**

| **SPM** | **Mean (pg/mL)** | **Median (pg/mL)** | **Min (pg/mL)** | **Max (pg/mL)** | **% Below**  **Limit of Detection** |
| --- | --- | --- | --- | --- | --- |
| 15-epi-LXA_4_ | 2.02 | 0.55 | 0.11 | 37.85 | 28 |
| 15-epi-LXB_4_ | 2.29 | 0.72 | 0.34 | 14.93 | 74 |
| 17R-RvD1 | 1.33 | 0.99 | 0.32 | 3.54 | 88 |
| 17R-RvD3 | 2.90 | 2.47 | 1.22 | 8.58 | 80 |
| 20-OH-LTB_4_ | NA | NA | NA | NA | 100 |
| 4S,14S-diHDHA | 3.41 | 2.74 | 0.22 | 12.35 | 66 |
| 5S,15S-diHETE | 9.41 | 2.99 | 0.62 | 139.29 | 48 |
| 7S,14S-diHDHA | 6.39 | 1.35 | 0.42 | 56.15 | 78 |
| 7S,14S-diHDPA | 2.16 | 0.51 | 0.29 | 13.45 | 82 |
| LTB_4_ | 3.72 | 0.43 | 0.12 | 121.23 | 26 |
| LXA_4_ | 1.33 | 0.66 | 0.13 | 7.87 | 62 |
| LXB_4_ | 1.88 | 1.38 | 0.53 | 7.27 | 72 |
| MaR1. | 0.81 | 0.74 | 0.37 | 1.68 | 80 |
| MaR1_n-3 DPA_ | 0.93 | 0.53 | 0.32 | 2.70 | 86 |
| PD1 | 5.95 | 3.82 | 1.97 | 35.39 | 44 |
| PDX | 6.92 | 0.65 | 0.13 | 136.70 | 54 |
| PGD_2_ | 1.92 | 0.77 | 0.15 | 36.69 | 24 |
| PGE_2_ | 1.46 | 0.69 | 0.19 | 9.54 | 30 |
| PGF_2a_ | 3.29 | 2.36 | 0.64 | 17.46 | 38 |
| RvD1 | 1.48 | 0.47 | 0.06 | 14.69 | 50 |
| RvD1_n-3 DPA_ | 0.74 | 0.64 | 0.11 | 2.75 | 64 |
| RvD2 | 1.23 | 0.40 | 0.19 | 7.40 | 78 |
| RvD2_n-3 DPA_ | 1.17 | 0.88 | 0.14 | 5.17 | 46 |
| RvD3 | 0.64 | 0.43 | 0.11 | 3.42 | 62 |
| RvD4 | 2.48 | 0.53 | 0.13 | 41.75 | 46 |
| RvD5 | 1.18 | 0.38 | 0.14 | 22.17 | 28 |
| RvD5_n-3 DPA_ | 0.71 | 0.22 | 0.12 | 5.13 | 58 |
| RvD6 | 2.45 | 0.95 | 0.14 | 16.95 | 78 |
| RvE1 | 3.47 | 1.62 | 0.25 | 21.57 | 72 |
| RvE2 | 40.85 | 0.64 | 0.23 | 195.59 | 90 |
| RvE3 | 4.28 | 2.29 | 0.12 | 37.63 | 52 |
| RvT1 | 2.56 | 1.44 | 0.31 | 17.84 | 62 |
| RvT2 | 0.39 | 0.26 | 0.15 | 0.88 | 92 |
| RvT3 | 0.43 | 0.32 | 0.11 | 0.95 | 84 |
| RvT4 | 1.09 | 0.49 | 0.12 | 9.44 | 24 |
| TxB_2_ | 20.71 | 9.54 | 0.32 | 178.69 | 54 |

**Supplementary Table 2. SPMs species and percentage of below limit of detection measurements.**

| **Cell populations** | **Comparison with NNC** | **FDR adjusted p-value** | | | |
| --- | --- | --- | --- | --- | --- |
|  |  | **GPR32** | | **GPR18** | |
|  |  | **Phenograph** | **FlowSOM** | **Phenograph** | **FlowSOM** |
| **MONOCYTES** | | | | | |
| C-M (HLA-DR-) | ALS | 0.011689182 | 0.011490214 | 0.613056671 | 0.093645906 |
| NC-M (HLA-DR-) | ALS | 0.011689182 | 0.966185131 | 0.527624268 | 0.820523327 |
| aCD11b+C-M  (HLA-DR-, CD11b Low) | ALS | 0.011689182 | 0.011490214 | 0.260931908 | 0.071090053 |
| aCD11b+IM-M  (HLA-DR-, CD11b Low) | ALS | 0.798988546 | 0.011490214 | 0.742752876 | 0.456202521 |
| aCD11b+ NC-M  (HLA-DR, CD11b Low) | ALS | 0.003658978 | 0.977523905 | 0.798988546 | 0.206728852 |
| C-M (HLA-DR-) | A-B | 0.027226777 | 0.038534285 | 0.922092294 | 0.093417409 |
| NC-M (HLA-DR-) | A-B | 0.027226777 |  | 0.753729688 | 0.495472704 |
| aCD11b+C-M  (HLA-DR-, CD11b Low) | A-B | 0.027226777 | 0.038534285 | 0.554517956 | 0.070234541 |
| aCD11b+IM-M  (HLA-DR-, CD11b Low) | A-B | 0.130447148 | 0.038534285 | 0.812805553 | 0.47374274 |
| aCD11b+ NC-M  (HLA-DR, CD11b Low) | A-B | 0.027226777 | 0.931132916 | 0.953608655 | 0.377196696 |
| C-M (HLA-DR-) | A-FB | 0.019548372 | 0.015619235 | 0.665249723 | 0.015619235 |
| IM-M (HLA-DR-) | A-FB | 0.049594449 | 0.0566484 | 0.654483224 | 0.060617889 |
| NC-M (HLA-DR-) | A-FB | 0.019548372 | 0.987407958 | 0.475090985 | 0.16463911 |
| aCD11b+C-M  (HLA-DR-, CD11b Low) | A-FB | 0.019548372 | 0.015619235 | 0.268354013 | 0.015619235 |
| aCD11b+IM-M  (HLA-DR-, CD11b Low) | A-FB | 0.099452445 | 0.016055089 | 0.575368943 | 0.086886367 |
| aCD11b+ NC-M  (HLA-DR, CD11b Low) | A-FB | 0.019306737 | 0.891060488 | 0.609544692 | 0.015619235 |
| C-M (HLA-DR-) | A-F | 0.002736274 | 0.003345218 | 0.801421644 | 0.056559017 |
| IM-M (HLA-DR-) | A-F | 0.011928932 | 0.012651041 | 0.801421644 | 0.16272871 |
| NC-M (HLA-DR-) | A-F | 0.002376127 | 0.941707484 | 0.574092566 | 0.621321034 |
| aCD11b+C-M  (HLA-DR-, CD11b Low) | A-F | 0.002376127 | 0.003187408 | 0.268430709 | 0.015247323 |
| aCD11b+IM-M  (HLA-DR-, CD11b Low) | A-F | 0.033714805 | 0.028573476 | 0.607811932 | 0.295515821 |
| aCD11b+ NC-M  (HLA-DR, CD11b Low) | A-F | 0.00115317 | 0.971758952 | 0.968711884 | 0.050025659 |
| **B CELL SUBSETS** | | | | | |
| US-B (CD24-) | ALS | 0.0223525 | 0.021749823 | 0.06499462 | 0.072135568 |
| B cells | A-B | 0.035907979 | 0.038684799 | 0.15447705 | 0.130543986 |
| N-B | A-B | 0.027226777 | 0.038684799 | 0.063239415 | 0.058849536 |
| US-B (CD24-) | A-B | 0.035907979 | 0.038534285 | 0.035907979 | 0.038534285 |
| LM-B | A-B | 0.959086146 | 0.940117806 | 0.027226777 | 0.038534285 |
| B cells | A-F | 0.002376127 | 0.003187408 | 0.084514488 | 0.074525942 |
| N-B | A-F | 0.043880799 | 0.045822046 | 0.033714805 | 0.028573476 |
| US-B (CD24-) | A-F | 0.008746812 | 0.007773118 | 0.004158086 | 0.004407216 |
| US-B (CD24+) | A-F | 0.002736274 | 0.003345218 | 0.087651307 | 0.077023071 |
| F-B | A-F | 0.032688279 | 0.02784841 | 0.043880799 | 0.045070069 |
| B cells | A-FB | 0.019548372 | 0.015619235 | 0.10729494 | 0.087142116 |
| N-B | A-FB | 0.019548372 | 0.015742016 | 0.065843698 | 0.057582207 |
| US-B (CD24-) | A-FB | 0.019548372 | 0.015619235 | 0.023724563 | 0.016055089 |
| US-B (CD24+) | A-FB | 0.019548372 | 0.015619235 | 0.099452445 | 0.079636099 |
| LM-B | A-FB | 0.945324181 | 0.945336419 | 0.049594449 | 0.044217961 |
| **T CELL SUBSETS** |  |  |  |  |  |
| M-Th  (CD25-, CD127+, FoxP3+) | ALS | 0.802502352 | 0.95247262 | 0.011689182 | 0.096815863 |
| N-Th  (CD25-, CD127+, FoxP3+) | ALS | 0.798988546 | NA | 0.015449154 | NA |
| M-Tcregs | ALS | 0.85129867 | NA | 0.033763211 | 0.072135568 |
| N-Tdpregs | ALS | NA | NA | 0.003658978 | 0.001493944 |
| M-Tdpregs | ALS | NA | 0.001493944 | NA | NA |
| N-Tc | ALS | NA | 0.524821265 | NA | 0.021749823 |
| N-Th (CD25-, CD127+, FoxP3+) | A-B | 0.857846018 | NA | 0.035907979 | NA |
| N-Th (CD25-, CD127-, FoxP3+) | A-B | 0.600079852 | 0.49408649 | 0.112811657 | 0.044006511 |
| M-Tdpregs | A-B | NA | 0.038534285 | NA | NA |
| M-Th (CD25-, CD127-, FoxP3+) | A-FB | 0.390656265 | NA | 0.049594449 | NA |
| M-Th (CD25-, CD127+, FoxP3+) | A-FB | 0.797324131 | 0.88608732 | 0.019548372 | 0.015619235 |
| N-Th (CD25-, CD127-, FoxP3+) | A-FB | 0.609544692 | 0.504313168 | 0.049594449 | 0.015619235 |
| N-Th (CD25-, CD127+, FoxP3+) | A-FB | 0.654483224 | NA | 0.036697267 | NA |
| T Cells | A-FB | 0.314251927 | 0.272071176 | 0.019548372 | 0.015619235 |
| Tregs | A-FB | 0.049594449 | NA | NA | NA |
| M-Th (CD25-, CD127-, FoxP3+) | A-F | 0.150344035 | NA | 0.020184985 | NA |
| M-Th (CD25-, CD127+, FoxP3-) | A-F | 0.831206621 | 0.979702341 | 0.033714805 | 0.180311941 |
| M-Th (CD25-, CD127+, FoxP3+) | A-F | 0.54175714 | NA | 0.00115317 | 0.004314454 |
| N-Th (CD25-, CD127-, FoxP3+) | A-F | 0.176751546 | 0.210366977 | 0.038264215 | 0.008549168 |
| N-Th (CD25-, CD127+, FoxP3+) | A-F | 0.441601706 | NA | 0.002376127 | NA |
| N-Tdpregs | A-F | NA | NA | 0.002376127 | 0.003187408 |
| T Cells | A-F | 0.187794543 | 0.187794543 | 0.013839805 | 0.012595272 |
| N-Tregs | A-F | 0.004238611 | NA | NA | NA |
| N-Tc | A-F | NA | 0.962669683 | NA | 0.00565355 |
| M-Tdnregs | A-F | NA | 0.003187408 | NA | NA |
| **SENESCENT T CELL SUBSETS** |  |  |  |  |  |
| LS-Tc | ALS | 0.885939462 | 0.966185131 | 0.003658978 | 0.012278642 |
| LS-Tc | A-B | 0.806178825 | 0.846095257 | 0.027226777 | 0.070234541 |
| LS-Tc | A-FB | 0.803271798 | 0.88608732 | 0.019548372 | 0.057582207 |
| LS-Tc | A-F | 0.894153139 | 0.835265577 | 0.043136092 | 0.10832406 |
| LS-Tc | A-SL | 0.957045521 | 0.9471416 | 0.002947659 | 0.041233636 |
| M-Th (CD27-, CD28+, KLRG1+, CCR7+) | A-SL | 0.416993612 | 0.53756651 | 0.029524164 | 0.097787222 |
| LS-Tc | A-S | 0.613341975 | 0.675434206 | 0.001632134 | 0.016988532 |
| M-Th (CD27-, CD28+, KLRG1-, CCR7+) | A-S | 0.608977874 | 0.597023905 | 0.042379595 | 0.095483068 |
| M-Th (CD27-, CD28+, KLRG1+, CCR7+) | A-S | 0.399760386 | 0.597023905 | 0.034285351 | 0.070722229 |
|  |  |  |  |  |  |

**Supplementary Table 3:** **Differential expression analysis by FlowSOM and Phenograph clustering.** Blue and red codes indicate a (statistically significant) resolvin receptors expression down and upregulation, respectively. Nomenclature and abbreviations are detailed in supplementary eTable 1. NA: not available.

| **Variable** | **Regression Coefficient** | **Regression Coefficient Robust Standard Error** | **Hazard Ratio (95% Confidence Interval)** | **P-Value** |
| --- | --- | --- | --- | --- |
| Baseline ALSFRS-R | 0.04 | 0.02 | 1.04 (1.08, 0.997) | 0.07 |
| Age at Onset (Years) | 0.04 | 0.02 | 1.04 (1.09, 1.00) | 0.05 |
| ΔFRS | 1.12 | 0.40 | 3.05 (6.72, 1.38) | 0.01 |

**Supplementary Table 4: Cox Proportional-Hazards Model Coefficients of pre-defined clinical independent variables.** Higher ALSFRS-R at baseline, Age at Onset, ΔFRS between onset and baseline are predictors of shorter survival.
